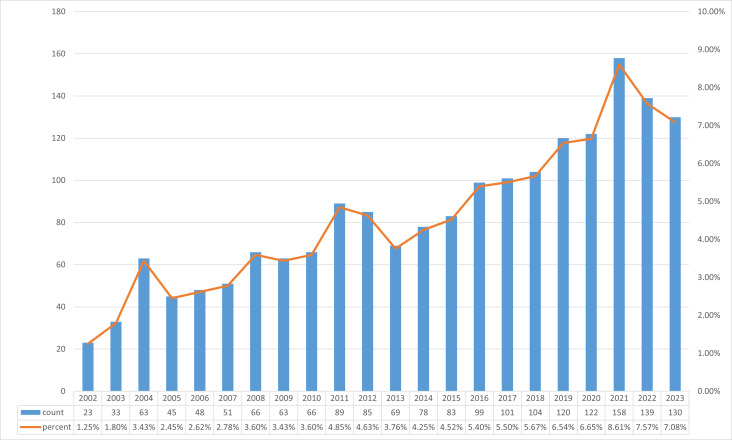# A bibliometric analysis of the effects of sex hormone profiles in women with polycystic ovary syndrome

**DOI:** 10.1097/MD.0000000000044965

**Published:** 2025-10-03

**Authors:** Bo Li, Shuang Li, Zibo Duan, Hui Yu, Yan Zhou, Xiaohua Lin

**Affiliations:** a Hebei Key Laboratory of Integrative Medicine on Liver-Kidney Patterns, Hebei University of Chinese Medicine, Shijiazhuang, Hebei, China; b Department of Gynecology, First Affiliated Hospital, Hebei University of Chinese Medicine, Shijiazhuang, Hebei, China; c Department of Gynecology, Handan First Hospital, Handan, Hebei, China.

**Keywords:** CiteSpace, bibliometrics, CiteSpace, PCOS, sex hormones, VOSViewer

## Abstract

**Background:**

Polycystic ovary syndrome (PCOS), a hormone-linked infertility disorder, severely impacts reproductive-aged women’s health and societal well-being.

**Methods:**

Bibliometric analysis via Web of Science (2013–2023) using CiteSpace, VOSviewer, and Bibliometric.

**Results:**

Analyzed 1835 studies from 80 countries, led by China/US. Research surged post-2017. *Journal of Gynecological Endocrinology* was most productive; *Fertility and Sterility* had the highest impact. Key topics: insulin resistance, obesity, testosterone. Legro (prolific) and Azziz (highly cited) dominated authorship.

**Conclusion:**

PCOS-sex hormone research holds growing scientific/social relevance. Current hotspots focus on metabolic-pathological mechanisms, guiding future investigations into personalized therapies and risk management.

## 1. Introduction

Hypoandrogenic anovulation, alternatively referred to as Stein-Leventhal syndrome, is a condition encompassing polycystic ovary syndrome. It is a metabolic endocrine disease of reproduction that combines psychological, reproductive, and metabolic disorders. From puberty to postmenopause, it is among the most prevalent inflammatory and oxidative diseases affecting women of childbearing age worldwide. Additionally, hyperandrogenism, ovulation disorders, and ovarian polycystic alterations are hallmarks of this condition. Clinical indications include the absence of a menstrual cycle, an irregular or prolonged menstrual cycle, or elevated testosterone levels. As clinical diagnostic criteria, the International Evidence-based Guidelines for the Assessment and Management of PCOS 2023 recommend the following: 1. Hyperandrogens of a clinical or biochemical nature; 2. Dysfunction of ovulation; and 3. Gynecological ultrasound assistance for polycystic ovaries. In order to exclude other potential etiologies of PCOS, a minimum of 2 of these 3 criteria must be met.^[1,2]^ PCOS is a persistent disease. While the exact etiology of PCOS remains unknown, environmental and genetic influences have been suggested as significant contributors. The pathophysiology of polycystic ovary syndrome is primarily attributed to the following factors: dysregulation of sex hormone secretion, chronic low-grade inflammation, hyperandrogenism, and insulin resistance. Certain factors have the potential to impede the growth of follicles and increase the likelihood of developing comorbidities such as endometrial cancer and type 2 diabetes mellitus.^[3]^ Epidemiological research confirms that a majority of women, exceeding 70%, suffer from infertility. It is believed that PCOS is among the leading causes of female infertility. The global prevalence of PCOS exhibits variation from 10 to 13%.^[4]^

Initiating and regulating the growth, function, and development of secondary sexual characteristics of reproductive organs, primarily estrogen, androgen, and progesterone, sex hormone is a type of hormone. Estrogens consisted of estriol (E3), estrone (E1), and estrol (E4), with 17β-estradiol being the most abundant among them.^[5]^ Progestin (P4) is the principal progesterone, whereas dihydrotestosterone (DHT), a more potent derivative of testosterone, is regarded as the principal androgen.^[6]^ Steroids are utilized in the synthesis of sex hormones from cholesterol. During this process, progesterone is converted to androgens, followed by estrogens. Progesterone and androgen synthesis are stimulated by (HSD) enzymes and Cytochrome P450 aromatase (CYP19A1) enzymes. The degradation of androgen into estradiol and estrogen is facilitated by the specific CYP enzyme aromatase, whereas 5α-reductase (5α-R) is responsible for the production of DHT. Diverse variables affect plasma concentrations of sex hormones, including sex, age, menstrual cycle stage, and pregnancy status; thus, individual variations are possible.^[7]^

Sex hormones, particularly androgens, have been linked to PCOS, according to numerous studies. follicles fail to develop and mature when sex hormone levels are abnormal. As a consequence, polycystic ovary syndrome may arise from the ovaries conserving an excessive quantity of embryonic follicles. Overproduction of luteinizing hormone (LH), which stimulates ovarian membrane cells to generate androgens, results from an excess of gonadotropin releasing hormone (GnRH) secretion.^[8]^ Failure to secrete follicle stimulating hormone results in follicular arrest, polycystic ovary morphology (PCOSM), hypoovulation, or anovulation, as opposed to follicle growth and maturation being inhibited. Simultaneously, follicular arrest induces the generation of a considerable quantity of preantral and small follicles, which subsequently stimulate heightened concentrations of anti-Mullerian hormone.^[9]^ This, in turn, stimulates further ovarian hyperandrogenism by increasing GnRH neuronal activity and directly stimulating the secretion of LH dependent on GnRH. Insulin resistance further results in hyperandrogenism, as hyperinsulinemia stimulates the secretion of GnRH and the production of androgens in ovarian membrane cells, while decreasing the production of sex hormone binding globulin.^[10]^

PCOS, which arises from atypical secretion of sex hormones, can give rise to various detrimental outcomes.^[11]^ These include but are not limited to obesity, metabolic syndrome, type 2 diabetes, infertility, and psychological disorders such as anxiety, depression, and low self-esteem. As of now, the prevailing therapeutic approaches for PCOS encompass interventions targeting insulin resistance, ovulation induction, weight loss, and abnormal sex hormone secretion, specifically excessive androgen secretion. To attain androgenic treatment, hormonal contraceptives, such as compound cyproterone acetate, are recommended. The drugs function through the inhibition of LH synthesis in the hypothalamic-pituitary-ovarian axis. This inhibition subsequently hinders theca cells’ ability to secrete significant quantities of androgen.^[12]^ In conclusion, the pathophysiology and physiology of PCOS, as well as treatment approaches, demonstrate that sex hormones have a significant impact on the disorder. As a result, research into the effects of sex hormones on PCOS may yield more dependable data that could lead to improved therapeutic approaches for this condition.

As interest in the effects of sex hormones on PCOS has grown in recent years, so has the body of literature. To enhance the organization of published studies and gain insights into forthcoming prominent trends, researchers must implement novel approaches for the review and organization of these studies.^[13]^ Bibliometrics is an exceptional approach that incorporates bibliometric mapping alongside bibliometric analysis. Bibliometric analysis employs this method to quantify the attributes of documents pertaining to a specific subject in order to uncover patterns within the body of scientific literature within a given field.^[14]^ This research employed bibliometric mapping to produce significant and comprehensible data regarding the impacts of sex hormones on PCOS through the subsequent means: 1) Applications in the medical field enable 1 to analyze a large number of publications and their output patterns at macro and micro levels^[15]^; 2) It can identify research hotspots, themes, legal developments, knowledge bases, research status, and research trends through a combination of qualitative and quantitative analysis.^[16]^ Bibliometric software, including CiteSpace, VOSViewer, and R-bibliometrix, is commonly employed to categorize and examine pertinent information pertaining to a specific field of study, including annual publication counts, nations, academic establishments, journals, authors, references, keywords, regions of high activity, and emerging themes. Based on the available literature, bibliometric analyses have been performed on the following topics: obesity and polycystic ovary syndrome^[17]^; insulin resistance and polycystic ovary syndrome^[18]^; infertility and polycystic ovary syndrome^[19]^; and polycystic ovary syndrome.^[20]^ However, there are no published bibliometric studies that examine the effects of sex hormones on PCOS. An examination was conducted using bibliometric analysis to discern and assess historical developments in the body of literature concerning sex hormones’ impact on PCOS, research topics (including countries and institutions, as well as prominent authors, journals, and publications). By identifying publication trends over time; major contributing countries and organizations; most influential authors and literature; previous research targets; current research status, priorities, and trends; and forecasts of future research hotspots, this endeavor seeks to generate new knowledge regarding the effects of sex hormones on PCOS. Furthermore, for academics, physicians, and medical students interested in the effects of sex hormones on PCOS, it provides a bibliometric report.

## 2. Materials and methods

### 2.1. Retrieval strategies and data cleaning

**Figure 1.**
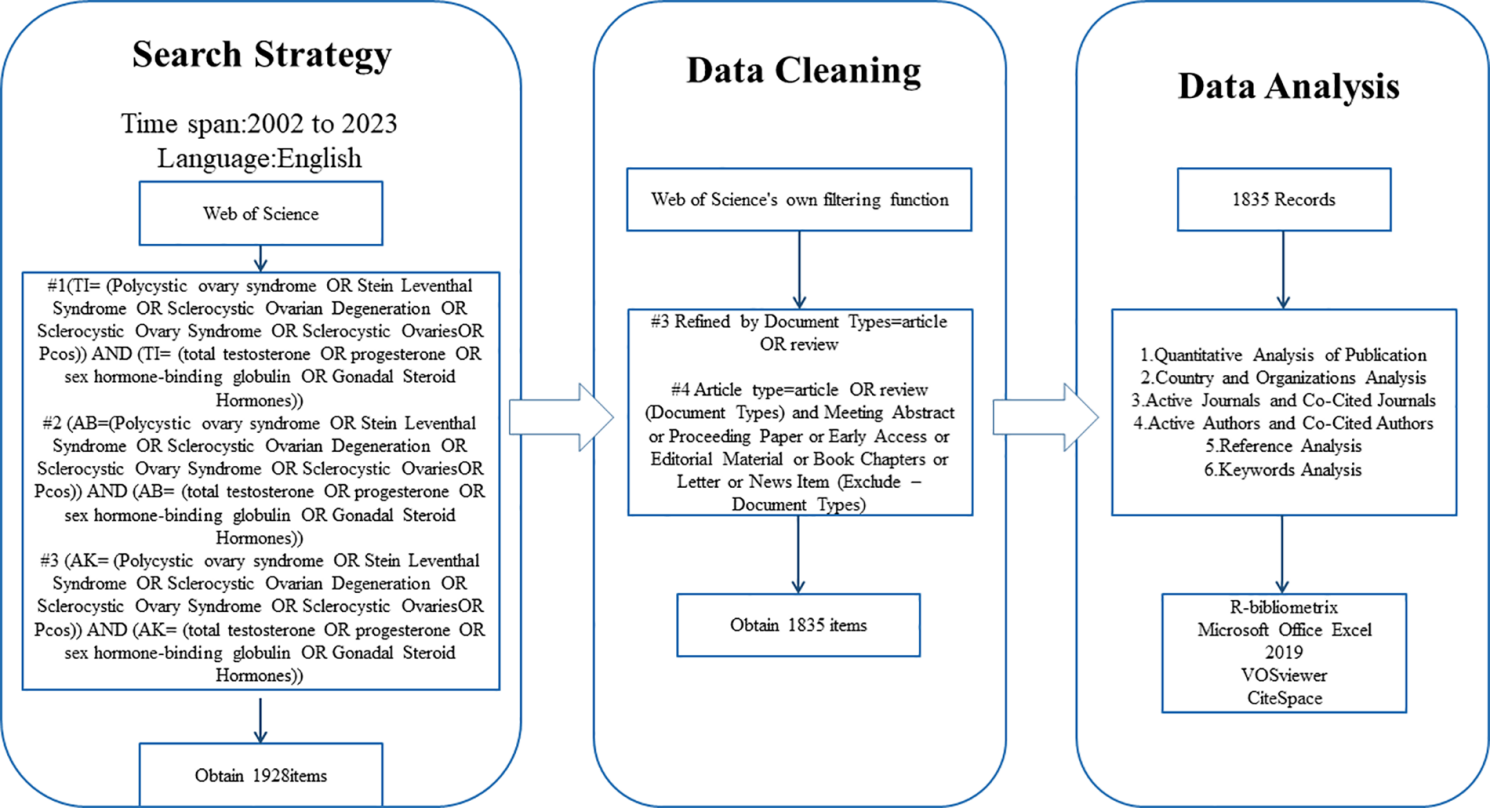
Data retrieval flowchart.

The search for the data utilized in this investigation commenced prior to November 24, 2023, and was conducted on the Web of Science Core Collection (WoSCC) database. Figure 1 illustrates the search formula. A cumulative of 1928 articles were identified. The projects underwent a refinement process based on the article types (meeting abstracts, meeting papers, editorial materials, chapters, early access, corrections, letters, data papers) using the screening function of Web of Science. This resulted in the deletion of 93 articles, leaving 1835 articles in the final compilation.

### 2.2. Data extraction

All data acquisition was conducted on November 24, 2023, via a search of WoSCC. Title, author, source, abstract, address, institution, document type, keywords, citations, and year of publication are all meticulously documented. The selected records are exported as “complete records and citations” for additional analysis. The text files were renamed “Download*.txt” in accordance with the compatibility of R-bibliometrix with *.txt versions and the fact that CiteSpace and VOSViewer exclusively accept *.txt versions.

We imported text files into CiteSpace, VOSViewer, and R-bibliometrix for the purpose of automating the analysis conducted by each software in this study.

### 2.3. Data analysis

Journal Citation Reports 2021 was utilized to calculate the quartiles and impact factors for the articles included in this study.

The following performance indicators were generated in this study using the open source tool R-bibliometrix (version 4.1) (https://www.bibliometrix.org), which is utilized for quantitative scienceometrics and bibliometrics research: total number of articles, types of articles, publications by country and institution, total number of references and co-citations, total number of keywords, total number of authors, and total number of co-cited authors.^[21]^ Then, the ten countries, organizations, journals, and authors with the greatest influence (as measured by the total number of publications), the ten most influential journals, authors, and citations (as measured by the number of co-citations of their publications), and the twenty most frequently occurring keywords were determined. R-bibliometrix is utilized for the 2 operations detailed below. 1) A network map depicting the global distribution of publications is created, with the degree of collaboration between nations denoted by the line thickness (a thicker line signifies greater cooperation); the degree of collaboration is quantified by the frequency of appearances of authors from distinct countries in the same publication. Furthermore, 2) an evaluation of the development of thematic trends and their correlation with hotspots and trends identified by the frequency of each time period is performed.

For the purpose of quantitatively analyzing published data, including the generation of visual charts and the calculation of percentages of total publications annually, Microsoft Office Excel (version 2019; Microsoft Corp., Redmond, WA, USA) is utilized.

VOSViewer (version 1.6.19) is software designed for text mining and bibliometric analysis.^[22]^ VOSViewer employs text mining to detect scientific information in publications, including co-occurrence analysis of references and keywords, countries and institutions, authors and co-cited authors, journals and co-cited journals, and references and keywords. Subsequently, visual similarity (VoS) mapping is utilized to generate bibliometric maps or landscapes.^[23]^ By importing data from a text file into VOSViewer, a visual bibliometric network is generated. A node represents an entity, such as a country, institution, journal, author, or keyword, on the VOSViewer map. The size and color of the nodes serve to denote the quantity and categorization of these entities. Co-referenced or collaborative the items are is indicated by the thickness of the lines that connect the nodes. In various visual networks, nodes or lines that share the same color signify greater relatedness, as in the case of keywords, co-cited journals, co-cited authors, and co-cited references. The colors of the nodes in the time scale visual network graph indicate the relative abundance of publications during the specified time period. These publications may include indicators of institutions, authors, countries, or journals.

Another bibliometric analysis and visualization program developed by Professor C. Chen, CiteSpace (version 6.2.R3) was utilized in this research to generate double map coverage and examine references experiencing significant citation spikes.^[24]^ Specific parameters are modified in CiteSpace: pruning (path finder), time segment (prior to November 24, 2023), and node type (references). The remaining parameters are left at their default values. Line thickness indicates co-occurrence intensity; colors denote distinct regions; a double map overlay illustrates the principal distribution fields of journals and co-cited journals, as well as the interrelationships among fields. The effect of a solitary citation over an extended duration is demonstrated by a robust surge in citations. For reference retrieval and visualization generation, utilize the default CiteSpace parameters; each bar symbolizes a year, years with significant citation surges are represented by red bars, influence strength is denoted by intensity, and publications with the longest burst durations are categorized according to endurance intensity.

## 3. Results

### 3.1. Quantitative analysis of publications

Prior to November 24, 2023, the search strategy yielded 1835 articles, comprising 1705 “articles” and 189 “reviews,” that delved into studies concerning the impact of sex hormones on polycystic ovary syndrome. On the basis of the annual growth rate of publications, the complete time period can be divided into 2 periods: Initial Period I (2002–2012) and Period II (2013–2023). As shown in Figure S1 (Supplemental Digital Content, https://links.lww.com/MD/Q207), the annual mean number of articles published in Period I is a relatively modest 63, suggesting that research into the effects of sex hormones on PCOS is in its nascent phase. While the number of articles published in Period I declines, it generally increases over time. The annual average number of publications began to increase substantially during Period II, reaching approximately 120. Although Period II is longer by twofold, it encompasses a greater volume of literature. A period of rapid development, Period II witnessed an annual increase in the number of publications; in 2021, there was a zenith of 158 publications devoted to the effects of sex hormones on polycystic ovary syndrome. This indicates that the academic community has devoted increasing attention to the effect of sex hormones on PCOS in recent years, demonstrating its significance.

### 3.2. Country and organization analysis

**Figure 2.**
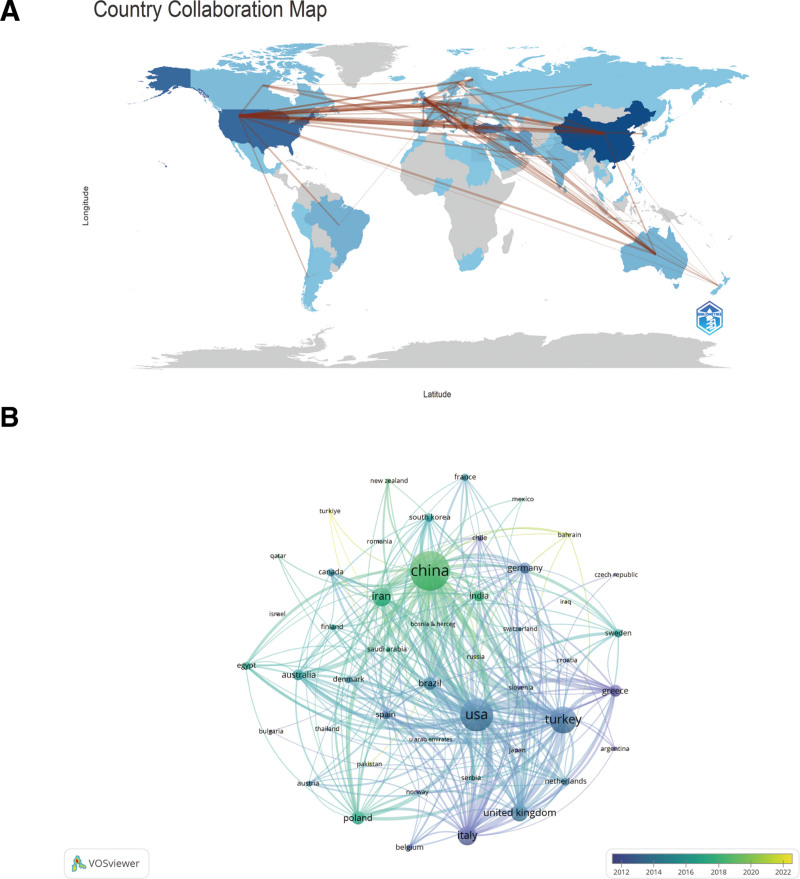
The global distribution (A) and intercountry visualization (B) of the effect of sex hormones on PCOS research. PCOS = polycystic ovary syndrome.

The sources of these publications are 2013 organizations and eighty countries. According to Table 1, the top ten countries are as follows: Asia (n = 3), North America (n = 1), Europe (n = 4), South America (n = 1), and Oceania (n = 1). Out of the countries included, China exhibited the maximum quantity of publications (n = 401, 21.85%), followed by Turkey (n = 216, 11.77%) and the United States (n = 296, 16.63%). The countries that concentrate their research efforts on investigating the impacts of sex hormones on PCOS produce an estimated half (49.75%) of all publications in this field. Following this, collaborative networks were constructed in each country, taking into account the number of publications and interrelationships among them (Fig. 2). We then filtered and visualized 47 countries (with a minimum of 5 publications). Notably, there exists a substantial degree of constructive collaboration among nations. The United States, for instance, maintains close working relationships with Japan, China, Turkey, Iran, and the United States; China, Britain, India, and Iran; and the United Kingdom, Turkey, Iran, the United States, and China. This observation indicates that developed and developing nations are already engaged in substantial collaboration.

**Table 1:** Ranked in the top 10 countries and institutions for research on the effects of sex hormones on PCOS.

Rank	Countries	Counts	Organizations	Counts
1	China (Asia)	401 (21.85%)	Heilongjiang University of Chinese Medicine (China)	34 (1.85%)
2	The United States (North America)	296 (16.13%)	Shanghai Jiao Tong University (China)	32 (1.74%)
3	Turkey (Asia)	216 (11.77%)	Tehran University of Medical Sciences (Iran)	29 (1.58%)
4	Iran (Asia)	126 (6.87%)	Aristotle University of Thessaloniki (Greece)	28 (1.53%)
5	Italy (Europe)	125 (6.81%)	China Medical University (China)	23 (1.25%)
6	The United Kingdom (Europe)	105 (5.72%)	Medical University of Silesia (Poland)	23 (1.25%)
7	Poland (Europe)	75 (4.09%)	Monash University (Australia)	23 (1.25%)
8	Brazil (South America)	68 (3.71%)	Shahid Beheshti University Medical Sciences (Iran)	23 (1.25%)
9	Greece (Europe)	62 (3.38%)	The Pennsylvania State University (The United States)	22 (1.20%)
10	Australia (Oceania)	55 (3.00%)	Shandong University (China)	22 (1.20%)

PCOS = polycystic ovary syndrome.

**Figure 3.**
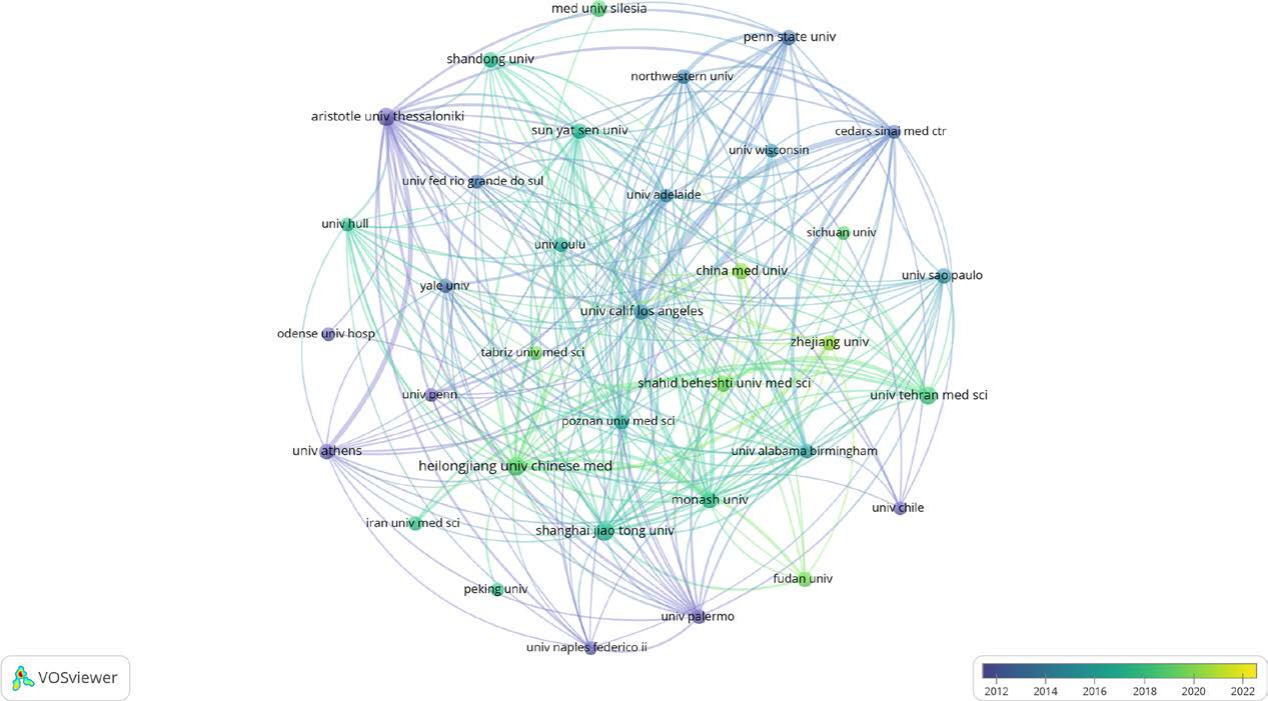
The visualization of organizations of the effect of sex hormones on PCOS research. PCOS = polycystic ovary syndrome.

The United States, China, Iran, Greece, Poland, and Poland comprise the top ten universities. Heilongjiang University of Traditional Chinese Medicine (China; n = 34, 1.85%), Shanghai Jiaotong University (China; n = 32, 1.74%), and Tehran Medical University (Iran; n = 29, 1.58%) were the 3 universities that accumulated the greatest number of publications (Table 1). A total of 35 organizations were chosen for visualization, with a publication threshold of 15 papers. Collaborative networks were then constructed among these organizations, with the connections between studies and the number of studies published by each organization serving as indicators (Fig. 3). Figure 3 illustrates the close collaboration between Heilongjiang University of Traditional Chinese Medicine (China), Zhejiang University (China), Fudan University (China), and Shanghai Jiaotong University (China); Tehran University Medical School (Iran) maintains strong partnerships with Zhejiang University (China), Fudan University (China), and Shanghai Jiaotong University (China); and Zhejiang University (China), Fudan University (China), and Heilongjiang University of Traditional Chinese Medicine (China). Nevertheless, it is worth noting that notwithstanding the substantial quantity of published papers, the initial 2 universities maintain a strong collaborative relationship, while the third university lacks such cooperation with the aforementioned 2.

### 3.3. Active journals and co-cited journals

**Figure 4.**
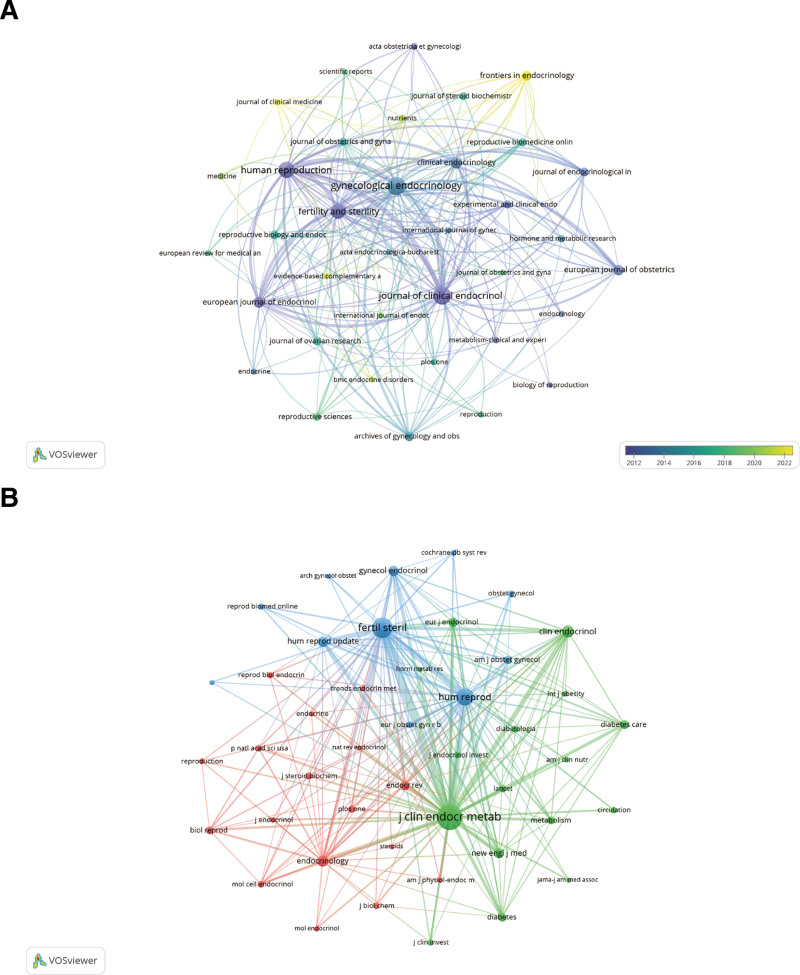
The visualization of journals (A) and co-cited journals (B) on research of the effect of sex hormones on PCOS research. PCOS = polycystic ovary syndrome.

A comprehensive collection of 1835 journal articles was devoted to the sex hormone PCOS. Journal of Clinical Electrophysiology and Metabolism (n = 120, 6.5%) and Human Reproduction (n = 102, 5.6%) published the remaining articles, in that order (Table 2). Gynecological Endocrinology published the plurality of articles (n = 135, 7.4%). After Human Reproduction (IF = 6.1) and Journal of Clinical Endocrinology & Metabolism (IF = 5.8), Fertility and Sterility (IF = 6.7) exhibited the highest IF among the top 10 journals (Table 2). A journal network map (Fig. 4A) was generated through the analysis of 36 journals that contained 10 articles or more. Figure 4A illustrates the highly active citation relationships that exist among journals including Human Reproduction, Journal of Clinical Endocrinology & Metabolism, Gynecological Endocrinology, and Fertility and Sterility.

**Table 2:** Top 10 journals and co-cited journals for research on the effects of sex hormones on PCOS.

Rank	Journals	Counts	IF	*Q*	Co-cited Journals	Co-citations	IF	*Q*
1	Gynecological Endocrinology	135 (7.4%)	2.0	Q3	Journal of Clinical Endocrinology Metabolism	9822	5.8	Q1
2	Journal of Clinical Endocrinology Metabolism	120 (6.5%)	5.8	Q1	Fertility and Sterility	5716	6.7	Q1
3	Human Reproduction	102 (5.6%)	6.1	Q1	Human Reproduction	4012	6.1	Q1
4	Fertility and Sterility	80 (4.4%)	6.7	Q1	Clinical Endocrinology	1713	3.2	Q3
5	Clinical Endocrinology	47 (2.6%)	3.2	Q3	Endocrinology	1471	4.2	Q2
6	European Journal of Endocrinology	43 (2.3%)	5.8	Q1	Gynecological Endocrinology	1307	2.0	Q3
7	European Journal of Obstetrics Gynecology and Reproductive Biology	37 (2.0%)	2.6	Q3	Human Reproduction Update	1217	13.3	Q1
8	Frontiers in Endocrinology	34 (1.9%)	5.2	Q1	New England Journal of Medicine	1077	158.5	Q1
9	Archives of Gynecology and Obstetrics	30 (1.6%)	2.6	Q3	Endocrine Reviews	966	20.3	Q1
10	Reproductive Biology and Endocrinology	27 (1.5%)	4.4	Q2	Journal of Clinical Endocrinology Metabolism	951	5.8	Q1

Co-citation refers to the simultaneous citation of 2 journals in the same article or articles. A knowledge kinship is established between 2 journals that are referenced by the same third journal. Journals that are influential in a particular subject can be identified through the use of journal co-citation analysis. According to the data presented in Table 2, 3 of the top ten co-cited journals have received citations exceeding 2000. Among these, the Journal of Clinical Electrophysiology and Metabolism is the most cited, with a total of 982 citations. Fertility and Sterility and Human Reproduction follow suit with 5716 and 4102 citations, respectively. Furthermore, Endocrine Reviews exhibited the lowest IF (20.3), while New England Journal of Medicine maintained the maximum IF (158.5). A network diagram of co-citations was subsequently constructed (Fig. 4B) using forty-four journals that met the minimum co-citation requirement of 250 articles. The Journal of Clinical Endocrinology & Metabolism exhibits robust co-citation relationships with several other journals, including Human Reproduction, Fertility and Sterility, and Clinical Endocrinology (Fig. 4B).

**Figure 5.**
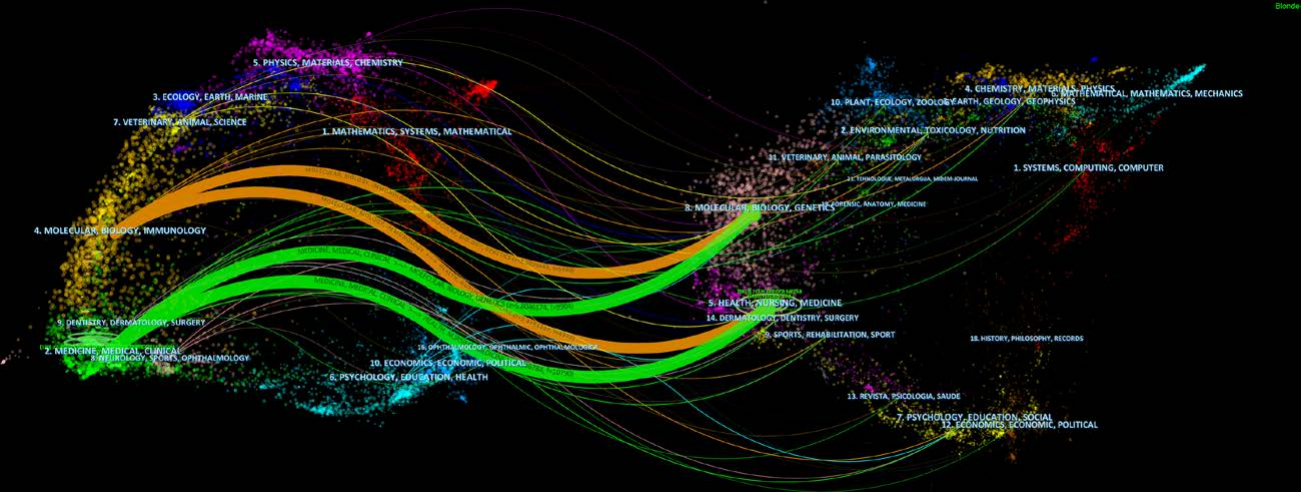
The dual-map overlay of journals and co-cited journals on the research off the effect of sex hormones on PCOS research. PCOS = polycystic ovary syndrome.

A double map overlay is employed in Figure 5 to illustrate citation relationships between journals, with co-cited journal clusters on the right and cited journal clusters on the left, as generated by CiteSpace. As illustrated in Figure 5, 2 reference paths exist. Research articles published in molecular/biology/immunology journals, which are predominantly cited by molecular/biology/genetics journals, are denoted by the orange path. The green path denotes scholarly articles that have been published in journals specializing in medicine, rehabilitation, sports, neurology, sports, and ophthalmology. These articles are predominantly cited in journals pertaining to health, nursing, medicine, dentistry, surgery, and sports/rehabilitation. Research on the impact of sex hormones on PCOS encompasses numerous disciplines, such as medicine, health, education, society, and psychology.

### 3.4. Active authors and co-cited authors

**Figure 6.**
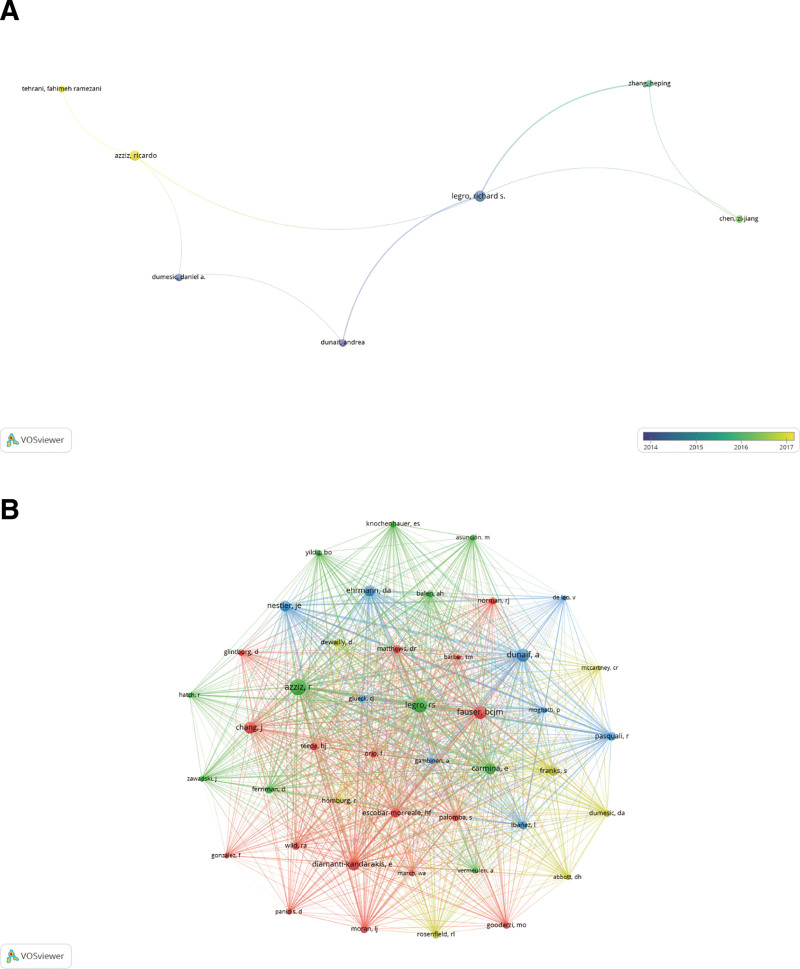
The visualization of authors (A) and co-cited authors (B) on research of the effect of sex hormones on PCOS research. PCOS = polycystic ovary syndrome.

The types of literature examined in this investigation were analyzed. A comprehensive assemblage of 8676 authors’ works examined the impact of sex hormones on PCOS. One of the top ten authors (Table 3) has authored over 25 articles, while 3 have published more than 15 articles. An author network was constructed by analyzing the works of eighteen authors who had published ten or more articles (Fig. 6A). A close collaboration has been established among several authors. As an illustration, Richard S Legro collaborates closely with Andrea Dunaif, Ricardo Azziz, Zijiang, Chen, Heping, Zhang, and others for approximately 5 years; Ricardo Azziz collaborates closely with Richard S Legro, Fahimeh Ramezani Tehrani, and Daniel A. Dumesic; and Heping, Zhang collaborates actively with Zijiang, Chen, Richard S Legro, and others. Three of the 27837 co-cited authors, Ricardo Azziz (979), Richard S. Legro (749), and Bart C. J. M. Fauser (629), accumulated more than 600 citations (Table 3). Regarding research into the effects of sex hormones on PCOS, these authors are respected authorities. Then, employing a minimum criterion of 100 co-citations, we determined that 44 authors satisfied this requirement; thus, a co-citation network graph was generated (Fig. 6B). Positive collaboration exists among co-citing authors, as illustrated in Figure 6B: Richard S. Legro and Enrico Carmina, Andrea Dunaif; Ricardo Azziz and Richard S. Legro, Evanthia Diamanti-Kandarakis; Richard S. Legro and Richard S. Carmina, Andrea Dunaif, and others. A robust collaboration exists among the frontrunners in this domain.

**Table 3:** Top 10 authors and coauthors in studies of the effects of sex hormones on PCOS.

Rank	Authors	Counts	Co-Cited Authors	Citations
1	Richard S. Legro	26	Ricardo Azziz	979
2	Ricardo Azziz	21	Richard S. Legro	749
3	Thozhukat Sathyapalan	16	Bart C. J. M. Fauser	629
4	Stephen L. Atkin	14	Andrea Dunaif	596
5	Dimitrios Panidis	13	Diamanti-Kandarakis E	528
6	Xiaoke Wu	13	Jeffrey Chang	508
7	Rui Alberto Ferriani	13	Enrico Carmina	469
8	Zi Jiang Chen	12	David A. Ehrmann	449
9	Andrea Dunaif	12	John E. Nestler	434
10	Daniel A. Dumesic	12	Stephen Franks	351

PCOS = polycystic ovary syndrome.

### 3.5. References analysis

**Figure 7.**
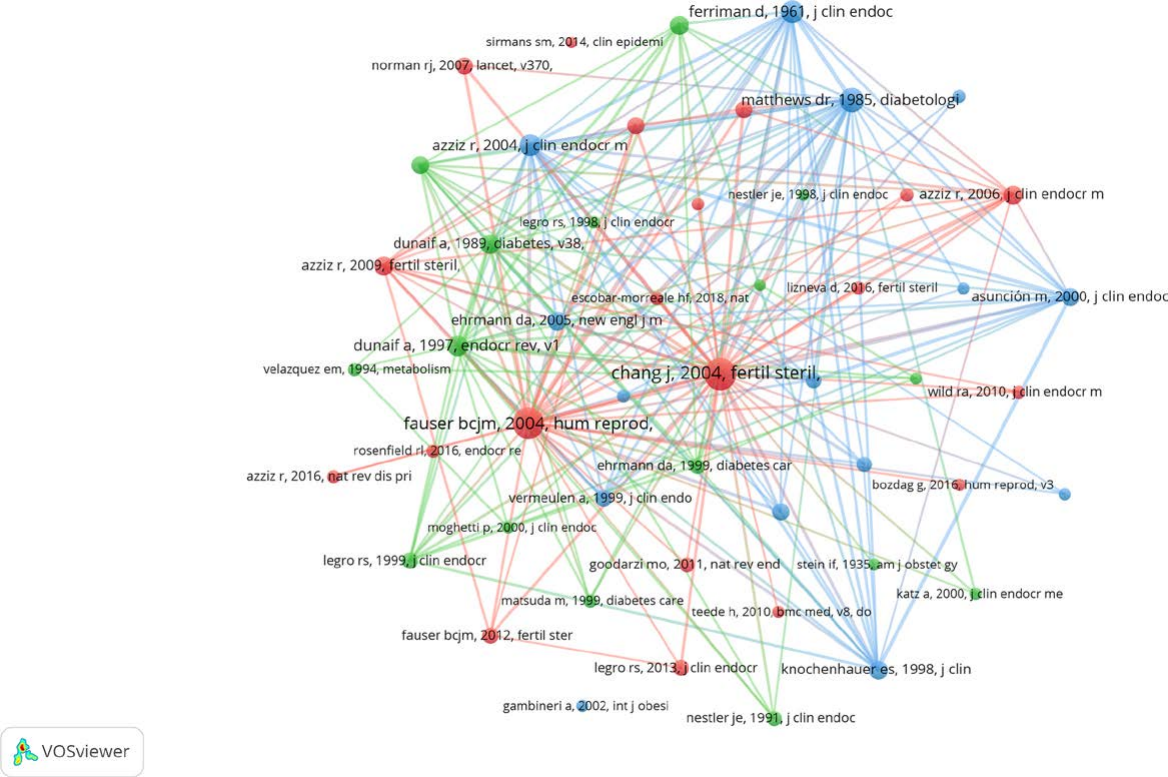
The visualization of co-cited references of the effect of sex hormones on PCOS research. PCOS = polycystic ovary syndrome.

The effects of sex hormones on PCOS have been the subject of 41088 citations in the past twenty-one years. As a result of being referenced in numerous studies, co-cited papers are regarded as the foundation of research in a given field.^[25]^ The article that has been cited the fewest number of 146 times is among the top ten most-cited (508 times), as shown in Table 4. A citation network was constructed by sifting through publications that had been cited 50% or more (Fig. 7). The reference relationships between “CHANG J, 2004, FERTIL STERIL and “FAUSER BCJM,2004, HUM REPROD,”^[1]^” “AZZIZ R,2004, CLIN ENDOCR M,” and “NORMAN RJ,2007, LANSET, V370” are evident, as illustrated in Figure 7.

**Table 4:** Top 10 references cited for studies on the effects of sex hormones on PCOS.

Rank	Co-cited reference	Citations
1	Chang J, 2004, Fertil Steril, V81, P19, DOI 10.1016/J.FERTNSTERT.2003.10.004	508
2	Fauser BCJM, 2004, Hum Reprod, V19, P41, DOI 10.1093/HUMREP/DEH098	469
3	Matthews DR, 1985, Diabetologia, V28, P412, DOI 10.1007/BF00280883	259
4	Ferriman D, 1961, J Clin Endocr Metab, V21, P1440, DOI 10.1210/JCEM-21-11-1440	218
5	Azziz R, 2004, J Clin Endocr Metab, V89, P2745, DOI 10.1210/JC.2003-032046	213
6	Dunaif A, 1997, Endocr Rev, V18, P774, DOI 10.1210/ER.18.6.774	191
7	Knochenhauer ES, 1998, J Clin Endocr Metab, V83, P3078, DOI 10.1210/JC.83.9.3078	152
8	Azziz R, 2009, Fertil Steril, V91, P456, DOI 10.1016/J.FERTNSTERT.2008.06.035	149
9	Franks S, 1995, New Engl J Med, V333, P853, DOI 10.1056/NEJM199509283331307	147
10	Azziz R, 2006, J Clin Endocr Metab, V91, P4237, DOI 10.1210/JC.2006-0178	146

PCOS = polycystic ovary syndrome.

**Figure 8.**
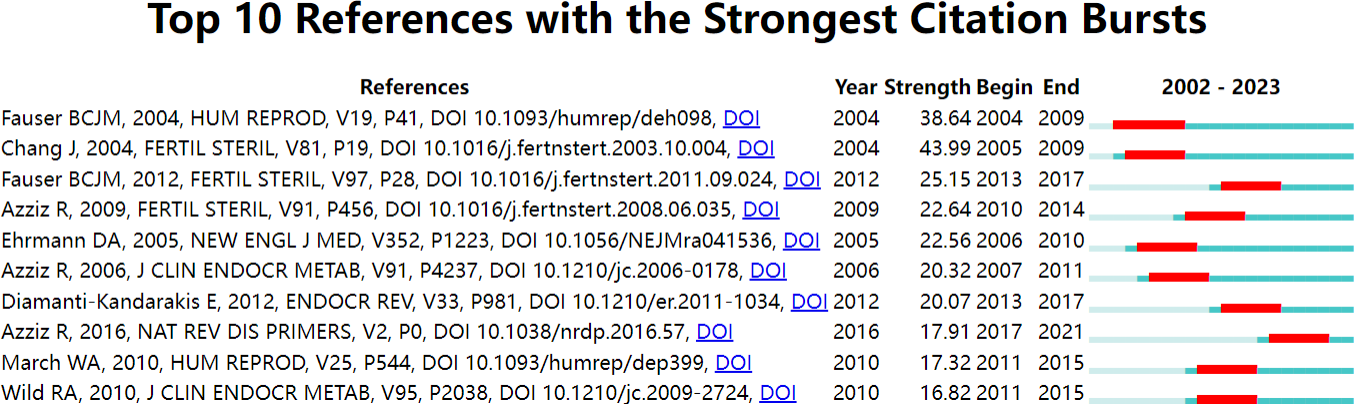
Top 10 references with strong citation bursts of the effect of sex hormones on PCOS research. PCOS = polycystic ovary syndrome.

CiteSpace was employed to examine substantial surges in citations, yielding a total of ten articles. Each bar corresponds to a year, as illustrated in Figure 8. Citation surge years are denoted by red bars. Beginning in 2004 and continuing through 2017, the quantity of citations increased. A abrupt increase in intensity varied between 16.82 and 43.99 years, whereas the sustained intensity of these ten articles ranged from 5 to 6 years. “Revised 2003 consensus on diagnostic criteria and long-term health risks associated with polycystic ovary syndrome” is the title of the Fertility and Sterility article that experienced the most significant increase in citations (strength = 43.99).

The primary findings of these ten articles, which are presented in Table 5 in the same order as the articles in Figure 8, are summarized.

**Table 5:** Major findings from 10 references with strong citation bursts.

Rank	Title	Authors	Strength	Main research results
1	Revised 2003 consensus on diagnostic criteria and long-term health risks related to polycystic ovary syndrome	Rotterdam ESHRE/ASRM-Sponsored PCOS consensus workshop group	38.64	PCOS is a syndrome of ovarian dysfunction along with the cardinal features hyperandrogenism and polycystic ovary (PCO) morphology.^[26]^
2	Revised 2003 consensus on diagnostic criteria and long-term health risks related to polycystic ovary syndrome	Rotterdam ESHRE/ASRM-Sponsored PCOS consensus workshop group	43.99	Insulin resistance and elevated serum LH levels are common features in PCOS. PCOS is associated with an increased risk of type 2 diabetes and cardiovascular events.^[27]^
3	Consensus on women’s health aspects of PCOS: the Amsterdam ESHRE/ASRM-Sponsored 3rd PCOS Consensus Workshop Group	Bart C J M Fauser 1, Basil C Tarlatzis, Robert W Rebar	25.15	Two widely cited previous ESHRE/ASRM-sponsored PCOS consensus workshops focused on diagnosis (published in 2004) and infertility management (published in 2008), respectively. The present third PCOS consensus report summarizes current knowledge and identifies knowledge gaps regarding various women’s health aspects of PCOS.^[28]^
4	The androgen excess and PCOS Society criteria for the polycystic ovary syndrome: the complete task force report	Ricardo Azziz 1, Enrico Carmina, Didier Dewailly	22.64	PCOS should be defined by the presence of hyperandrogenism (clinical and/or biochemical), ovarian dysfunction (oligo-anovulation and/or polycystic ovaries), and the exclusion of related disorders.^[29]^
5	The prevalence and features of the polycystic ovary syndrome in an unselected population	Ricardo Azziz 1, Keslie S Woods, Rosario Reyna	22.56	data from a large representative unselected population support the concept that PCOS is the most common endocrine abnormality of reproductive-aged women in the United States.^[30]^
6	Positions statement: criteria for defining polycystic ovary syndrome as a predominantly hyperandrogenic syndrome: an Androgen Excess Society guideline	Ricardo Azziz, Enrico Carmina, Didier Dewailly	20.32	A principal conclusion was that PCOS should be first considered a disorder of androgen excess or hyperandrogenism.^[31]^
7	Insulin resistance and the polycystic ovary syndrome revisited: An update on mechanisms and implications	Evanthia Diamanti-Kandarakis, Andrea Dunaif	20.07	some groups of lean affected women may have normal insulin sensitivity. There is a postbinding defect in receptor signaling likely due to increased receptor and insulin receptor substrate-1 serine phosphorylation that selectively affects metabolic, These insights have been directly translated into a novel therapy for PCOS with insulin-sensitizing drugs.^[32]^
8	Polycystic ovary syndrome	Ricardo Azziz, Enrico Carmina, ZiJiang Chen	17.91	This Primer summarizes the current state of knowledge regarding the epidemiology, mechanisms and pathophysiology, diagnosis, screening and prevention, management and future investigational directions of the disorder.^[33]^
9	The prevalence of polycystic ovary syndrome in a community sample assessed under contrasting diagnostic criteria	Wendy A. March, Vivienne M. Moore, Kristyn J. Willson,	17.32	The Rotterdam and AES prevalence estimates were up to twice that obtained with the NIH criteria in this, as well other prevalence studies. In addition, this study also draws attention to the issue of many women with PCOS in the community remaining undiagnosed.^[34]^
10	Assessment of cardiovascular risk and prevention of cardiovascular disease in women with the polycystic ovary syndrome: A consensus statement by the Androgen Excess and Polycystic Ovary Syndrome (AE-PCOS) Society	Robert A. Wild, Enrico Carmina, Evanthia Diamanti-Kandarakis	16.82	Women with PCOS with obesity, cigarette smoking, dyslipidemia, hypertension, impaired glucose tolerance, and subclinical vascular disease are at risk, whereas those with metabolic syndrome and/or type 2 diabetes mellitus are at high risk for CVD.^[35]^

AES = Androgen Excess Society, CVD = cardiovascular disease, NIH = National Institutes of Health.

### 3.6. Keywords analysis

**Figure 9.**
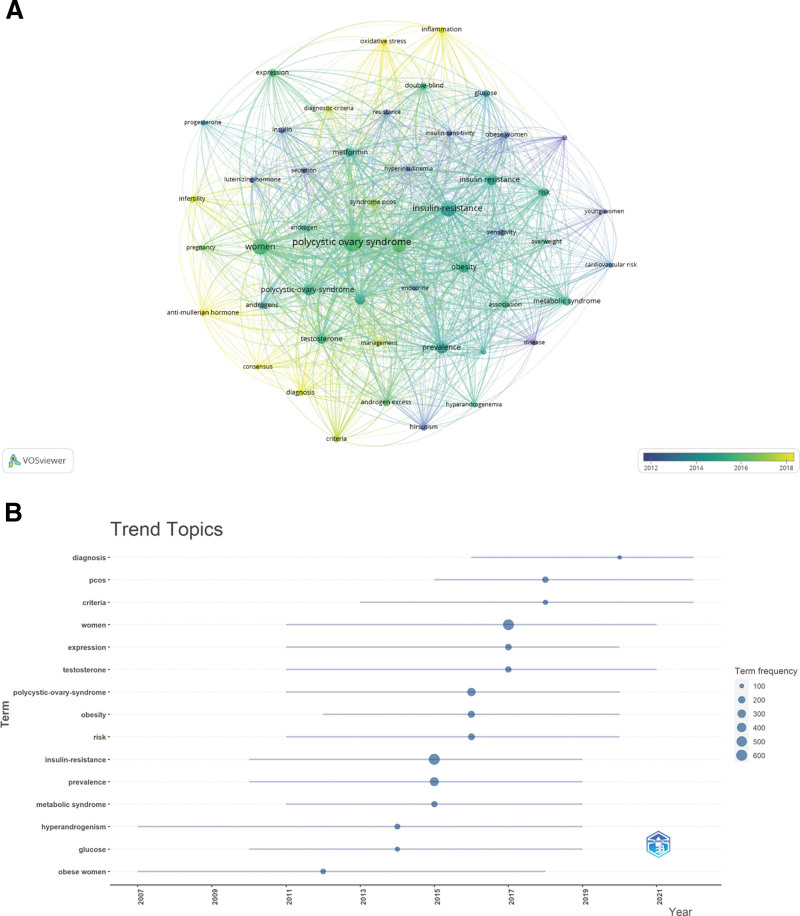
The visualization of frequency keywords (A) and trend topic analysis (B) on research of the effect of sex hormones on PCOS research. PCOS = polycystic ovary syndrome.

Rapidly identifying frontiers and concentrations of sex hormone impact on PCOS across multiple time periods is possible via keyword analysis. Included in Table 6 are the twenty most frequently occurring keywords in research on the effects of sex hormones on PCOS. Weight gain is the most frequently utilized keyword among these, excluding insulin resistance, women, prevalence, and polycystic ovary syndrome. This signifies the primary focus of research regarding the impact of sex hormones on PCOS. Visual cluster analysis was conducted on keywords that appeared in Figure 9A and contained a minimum of 60 occurrences. By examining the thickness of the lines connecting the nodes, it is possible to discern the intensity of the links between keywords. The outcomes are depicted in the form of a network graph, which yields 3 clusters that correspond to 3 distinct research directions. Polycystic ovary syndrome, hyperandrogenism, and rotund women are categories of keywords found in the red cluster. Insulin resistance, metabolic syndrome, and obesity are terms occurring in the green cluster. Blue clusters encompass the following keywords: testerone, diagnostic, criteria, and consensus. The pathological mechanism of PCOS, complications associated with PCOS, and diagnostic criteria pertaining to sex hormones in PCOS are delineated in the red, green, and blue clusters, respectively. The pathological mechanism and complications of PCOS resulting from abnormal sex hormone secretion, as well as diagnostic criteria, are thus the primary research foci of the influence of sex hormones on PCOS.

**Table 6:** Top 20 keywords in studies of the effects of sex hormones on PCOS.

Rank	Words	Counts	Rank	Words	Counts
1	Insulin-resistance	601	11	Syndrome PCOS	128
2	Women	571	12	Association	124
3	Prevalence	350	13	Hyperandrogenism	124
4	Polycysticovarysyndrome	300	14	Obese women	119
5	Obesity	190	15	Androgen excess	117
6	Risk	180	16	Metformin	112
7	Expression	161	17	Criteria	110
8	Testosterone	150	18	Glucose	106
9	PCOS	146	19	Double-blind	100
10	Metabolic syndrome	140	20	Diagnosis	97

PCOS = polycystic ovary syndrome.

A topic trend analysis was conducted using the R-bibliometrix; the results are depicted in Figure 9B in accordance with the time segments of each trending topic. Diagnosis is a topic from 2016 to 2022, while hyperandrogenism and insulin resistance are topics of interest from 2007 to 2019, metabolic syndrome is a popular topic from 2011 to 2019, and obesity is a trending topic from 2012 to 2020. On the basis of the aforementioned topic trends, it is possible to conclude that earlier research on the effects of sex hormones on PCOS was preoccupied with the pathological mechanism and complications, whereas more recent research has been preoccupied with the development of diagnostic criteria for PCOS.

## 4. Discussion

### 4.1. General information

Sex hormones and PCOS were the subjects of this investigation. 632 articles were published during the initial period (2002–2012), or an average of 63 per year. The present era is still in its nascent phases of research in the field, as shown in Figure S1 (Supplemental Digital Content, https://links.lww.com/MD/Q207), but the volume of research content is progressively expanding. Twelve hundred 3 articles were published annually on average for ten years during the second period (2013–2023), which is roughly double the number published during the first period (120 articles per year). This indicates that scholarly investigations into the impacts of sex hormones on PCOS are expanding exponentially throughout the current decade, thereby indicating a heightened interest in the subject matter.

The foremost countries worldwide in the investigation of the effects of sex hormones on PCOS are China, the United States, and Turkey. Collectively, they contribute to around half of the global articles on this subject (n = 401, 21.85%), while the United States contributes (n = 296, 16.63%). Four of the top ten institutions in this discipline are situated in China, while the remaining 2 are in Iran. Close international cooperation exists, particularly between China and the United States, India and Iran, Turkey, and Britain. Furthermore, research institutions from different nations engage in close collaboration. Notably, Shanghai Jiaotong University maintains a strong partnership with Zhejiang University, Fudan University, and Heilongjiang University of Traditional Chinese Medicine; Zhejiang University, Fudan University, and Shanghai Jiaotong University all collaborate closely with Shanghai Jiaotong University; and Tehran University School of Medical Sciences maintains a close partnership with Tehran University. The country analysis reveals that developed and developing nations already collaborate closely, but the analysis of the top 10 institutions reveals that intra-country cooperation, such as that of China, continues to dominate inter-agency cooperation. This indicates that there is no global network of partnerships and international cooperation remains inadequate, neither of which are conducive to the advancement of research concerning the effects of sex hormones on polycystic ovary syndrome. Therefore, to facilitate the development of a global collaborative network for research on the effects of sex hormones on PCOS, we strongly advise more extensive and detailed collaborative research between academic institutions from various nations.

The majority of studies examining the effects of sex hormones on polycystic ovary syndrome were published in Gynecological Endocrinology (Q3), the most frequently cited journal in the field (n = 135 (7.4%), IF = 6.313; n = 135; 7.4%); however, it does not have the highest impact factor. The journal that generated the greatest impact factor (IF = 6.7, Q1) among those that examined the effects of sex hormones on PCOS was Fertility and Sterility (n = 80, 4.4%). Nevertheless, the disparities in impact among these journals are negligible. Overall, the Journal of Clinical Endocrinology & Metabolism is the most influential and most cited journal. In conjunction with timeline analysis, Frontiers In Endocrinology is the primary journal for current research on the effects of sex hormones on PCOS. This suggests that an increasing number of journals are focusing on research concerning the effects of reproductive hormones on PCOS. Q1 journals, which furnish studies on the effects of sex hormones on PCOS with high-quality literature support, comprise the majority of the co-cited journals.

Author analysis determined that Richard S. Legro (n = 26) rated first in terms of the quantity of articles published. According to the article by Richard S. Legro, PCOS is a psychological, metabolic, and reproductive disorder with exceedingly complex causes that affects the entire life cycle. The factors that contribute to this condition are genetic and epigenetic predisposition, insulin resistance and mechanisms associated with obesity, as well as dysfunction in the hypothalamus and ovaries.^[26]^ An important characteristic of polycystic ovary syndrome is the excessive production of androgens by the ovaries. The condition is associated with an increased risk of developing type 2 diabetes, gestational diabetes and other complications related to pregnancy, venous thromboembolism, cerebrovascular and cardiovascular events, and endometrial cancer.^[27]^ A healthy, well-balanced diet and regular exercise are components of the lifestyle modification and medical management strategies. To prevent excessive weight gain, the lifestyle modifications consist of metformin, which improves metabolic profiles and insulin resistance; combination oral contraceptives, which address both hyperandrogenism and menstrual cycle regulation; and antiandrogens, when required, to treat refractory hyperandrogenism.^[28]^ Overall, the study by Richard S. Legro analyzes and clarifies the function of sex hormones (androgens) in the pathogenesis, consequences, and treatment of PCOS. This has significantly advanced the study of reproductive hormones in individuals with PCOS.

The author with the highest number of citations is Ricardo Azziz, with 979. Richard S. Legro follows with 749 citations, then Bart C. J. M. Fauser with 629 citations, and finally Andrea Dunaif with 596 citations. Ricardo Azziz observed in 2002 that insulin resistance affects 50 to 70% of women with PCOS and 100 to 80% of individuals with type 2 diabetes, with degrees ranging from 80 to 100%. Insulin resistance and its secondary hyperinsulinemia seem to be the cause of a number of endocrine symptoms in the majority of patients with PCOS, suggesting a possible association between the 2 conditions.^[29]^ Ricardo Azziz postulated prospectively the following year that “excessive androgen levels are a crucial factor in polycystic ovary syndrome.”^[30]^ The foundation for the effects of reproductive hormones on PCOS was established by this theory. Despite the fact that few individuals contemplate the possibility that PCOS does not exist, a subsequent review published by Ricardo Azziz in 2006 asserts that PCOS should fundamentally be considered as a disorder characterized by androgen excess.^[31]^ Azziz 2008 demonstration that alleles containing fewer CAG repeats exhibited greater androgen receptor (AR) activity indicated that genetic modifications in androgen sensitivity might be a factor in polycystic ovary syndrome.^[32]^ Consequently, scholarly investigations centered on the pathological determinants of PCOS, such as hyperinsulinemia and insulin resistance. It is noteworthy that Ricardo Azziz employed logistic regression model analysis in a 2011 article to establish a statistically significant correlation between PCOS and women who have high personal education or low parental education. He concluded that women who have children with Socioeconomic Status (SES) in their lives face an elevated risk of developing PCOS, thereby implying that education may have an impact on the prevalence of PCOS.^[33]^ Ricardo Azziz noted in a 2013 article that patients with polycystic ovary syndrome have an elevated risk of developing gestational hypertension and diabetes.^[34]^ Young women with PCOS had substantially greater arterial intima media thickness measurements than young women without PCOS, according to a 2014 article by Ricardo Azziz. Based on this finding, women with PCOS had an increased risk of developing subclinical cardiovascular disease.^[35]^ Androgen excess is a prominent characteristic of polycystic ovary syndrome; consequently, clinical management for this condition may involve androgen suppression (hormonal combination contraceptives), androgen blockers (including AR blockers and 5-α-reductase inhibitors), or a hybrid approach involving both (Azziz, 2016). It is advisable to integrate cosmetic treatments, such as targeted therapies for hirsutism and acne, with pharmacological treatments.^[36]^ PCOS substantially increases the risk of type 2 diabetes, pregnancy-related cardiovascular events, and endometrial cancer, according to a 2018 review by Ricardo Azziz.^[37]^ Recent years have seen a transition in Ricardo Azziz research emphasis from the pathogenesis of PCOS to its adverse effects and treatment. Ricardo Azziz, one of the foremost authorities on the academic implications of sex hormones on PCOS, emphasized the trajectory of the current body of research, which is to concentrate on the detrimental consequences of PCOS and potential therapeutic interventions.

On the references, we initially conducted co-reference analysis. The document with the highest number of citations, 508, was the Revised 2003 consensus on diagnostic criteria and long-term health hazards associated with PCOS. Diagnostic criteria should be revised in light of the consensus that PCOS encompasses a more extensive spectrum of ovarian dysfunction signs and symptoms than was initially defined. The 2003 criteria (2 out of 3) replaced the 1990 diagnostic criteria (including 1 and 2): “1. Chronic anovulation, 2. Clinical and/or biochemical signs of hyperandrogenism, and exclusion of other causes” (as above): “1. Hypoovulation and/or anovulation; 2. Clinical and/or biochemical signs of hyperandrogenism; 3. Polycystic ovary; and exclusion of other causes (congenital adrenal hyperplasia, androgen-secreting tumors, Cushing syndrome); This revised criteria establishes the groundwork for a more precise diagnosis of PCOS. Additionally, it establishes a body of literature that other researchers can utilize to investigate the health implications of polycystic ovary syndrome. Furthermore, it describes the essential knowledge that novice medical students and scholars must possess.

Citations to articles that have experienced citation surges signal the most significant contemporary subjects within the research domain, given their extensive utilization as citations in recent times. Based on the results of the main content analysis of citations with citation burst (Table 5), the primary research areas concerning the impact of sex hormones on PCOS are as follows: the pathological mechanism underlying this influence; the epidemiology of PCOS; strategies for preventing, managing, and treating PCOS; and the adverse effects of PCOS on the cardiovascular and endocrine systems. As indicated by the 2-map overlay analysis, research pertaining to the impact of sex hormones on PCOS encompasses an extensive domain. Consequently, interdisciplinary interactions that collectively advance research necessitate the collaboration of numerous research fields.

### 4.2. Hot spots and trends

A trend analysis of keywords and topics facilitates the rapid elucidation of the intricacies of the direction of research in a particular field. Metabolic syndrome (n = 140), insulin resistance (n = 601), obesity (n = 190), and hyperandrogenism (n = 140) were frequently used keywords, as determined by bibliometric analysis (Table 6, Figure 9B). According to Figure 9B, the following conditions exhibited a notable trend: hypertension from 2007 to 2019, insulin resistance from 2010 to 2019, metabolic syndrome from 2011 to 2019, and insulin resistance from 2012 to 2020.

The keywords may be categorized into 3 distinct clusters: the complications associated with PCOS are denoted by the green cluster, while the pathological mechanism of PCOS is represented by the red cluster. In PCOS, the diagnostic criteria for reproductive hormones are represented by the blue clusters. Consequentially, the following are current developments in the investigation of the impact of reproductive hormones on hypertension.

### 4.3. Diagnostic criteria for PCOS

As of the present moment, 4 diagnostic consensuses have gained international recognition, one of which is the National Institutes of Health (NIH). Committee for European Society for Reproductive and Embryological Medicine, National Institutes of Health (NIH) American Society for Reproductive Medicine (ASRM) and European Society of Human Reproduction and Embryology (ESHRE) The ASRM Rotterdam criteria, the American Society for Androgen Excess (AES) AES criteria, and the American Endocrine Society’s (2013) guidelines for the diagnosis and treatment of PCOS.

1. 1990 NH criteria: clinical manifestations of hyperandrogenism and/or hyperandrogenism; chronic anovulation; except for other related diseases with these symptoms, such as hyperprolactinemia and thyroid diseases, congenital adrenal hyperplasia. The diagnostic criteria were the first widely accepted diagnostic criteria for PCOS developed in 1990 by the United States Health Organization/United States Organization for Child Health and Human Development (NIH/NICHD), which laid the foundation for the diagnosis of PCOS. It is important to note that this diagnostic criterion does not provide specific explanations for each criterion and does not include the key feature of polycystic ovarian changes. Therefore, this diagnostic criterion has great limitations in the diagnosis of PCOS and has been controversial internationally since it was proposed.
2. 2003 Criteria for Rotterdam: clinical presentations and/or biochemical alterations associated with hyperandrogenism; sporadic ovulation or anovulation; prostate cancer on ultrasound – detection of at least 12 follicles measuring 2 to 9 mm in diameter on the same section and an ovarian volume of 10 mL (ovarian volume = 0.5 × length × breadth × thickness).^[38]^ The European Society for Human Reproduction and Embryology (ESHRE) and the ASRM collaboratively introduced these criteria in 2003. A diagnosis can be established with the fulfillment of only 2 out of the 3 criteria for aberrant ovulation – high androgens and PCO – according to these criteria. As an adjunct to the NH criteria, PCO was incorporated, thereby enhancing the diagnostic criteria for PCOS and furnishing more robust guidance for clinical diagnosis. However, due to the overly broad diagnostic criteria, certain women who do not have endocrine abnormalities or fertility disorders may be erroneously diagnosed with PCOS and subsequently prescribed unsuitable treatment. Nonetheless, it continues to be the diagnostic standard most extensively employed on a global scale.
3. Criteria for 2006 AES: the absence of PCO and/or hirsutism and/or hyperandrogenism; oligoovulation or anovulation; and/or hirsutism and/or hyperandrogenism.^[39]^ In 2006, the AES recommended this diagnostic criterion. B-ultrasound examiners’ assessments of ovarian polycystic disease and follicle size may be subject to variation due to the presence of such alterations in healthy women. Additionally, some patients do not exhibit the characteristic metabolic abnormalities of PCOS according to the new Rotterdam criteria diagnosis. The diagnostic significance of hyperandrogenism in PCOS is emphasized in the AES criteria, which require both biochemical/clinical hyperandrogenism and ovarian dysfunction (oligo-anovulation or PCOSM). Notably, hyperandrogenism alone cannot definitively diagnose PCOS; it must coexist with either ovulatory disorders (e.g., oligoovulation or anovulation) or ultrasonographic evidence of PCOM. This diagnostic framework expands the 1990 NIH criteria by incorporating PCOM as an independent criterion, aligning more closely with the Rotterdam consensus while maintaining a stronger emphasis on androgen excess compared to broader Rotterdam definitions.4. AES Guidelines for 2013: Clinical and/or biochemical indications of an androgen excess; ovulation that occurs infrequently or not at all; and ovarian polycystic alterations. In other words, the diagnostic criteria adhere to the 2003 Rotterdam diagnostic criteria, which provide exhaustive descriptions and definitions for each criterion. PCOS is diagnosed when 2 of the subsequent 3 criteria are met, while excluding analogous clinical manifestations that may be attributed to alternative disorders. This ongoing-use standard is an additional development of the Rotterdam standard.^[40]^

## 5. Pathological mechanisms and complications

### 5.1. Pathological mechanisms

Insulin resistance (IR) is characterized by compromised insulin sensitivity in target tissues, including liver, muscle, and adipose.^[41]^ The main feature of this condition is a diminished utilization of glucose, which is caused by impairments in the transmembrane glucose transport that are enabled by type 4 glucose transporters.^[42]^ IR is caused by postreceptor abnormalities resulting from the disruption of insulin receptor downstream signaling in PCOS. Consequently, the sensitivity and responsiveness of insulin-resistant tissues to insulin stimulation are diminished, with this consequence being particularly conspicuous among obese patients with PCOS.^[43]^ In 1980, it was first recognized that insulin resistance (IR) and PCOS could coexist. Studies at the time indicated that insulin hypersecretion was prevalent among women with PCOS, indicating a potential association.^[44]^ 75% of women diagnosed with PCOS exhibit insulin resistance, or diminished insulin sensitivity, as determined by the hyperinsulin-euglycemic clamp.^[33]^ The presence of insulin receptors in ovarian stromal cells and follicular cells indicates that insulin is a significant factor in ovarian function. Significant evidence supports the notion that insulin directly influences the production of steroid hormones and that insulin signaling pathways are crucial in regulating ovulation.^[45]^ Ovarian membranous cells serve as the principal locations for androgen synthesis. When functioning normally, insulin functions as an accessory gonadotropin through its binding to its specific receptor. This binding stimulates the secretion of LH from membranous cells, which subsequently triggers the synthesis of androgens and the luteinization of granulosa cells.^[46]^ Insulin resistance can arise from pathological circumstances, such as intestinal flora imbalances, alterations in environment, mood, and diet, as well as abnormal levels of metabolites produced by bacteria. These factors disrupt insulin receptor signaling, which subsequently induces chronic low-grade inflammation and immune system dysfunction.^[47]^ When insulin resistance develops and insulin accumulates in the body, hyperinsulinemia results. Excessive secretion of GnRH can result from hyperinsulinemia; this, in turn, stimulates ovarian membrane cells (In the follicular structure, theca cells are stromal cells located in the outer layer of the follicle (FL), adjacent to the exterior of granulosa cells and separated by the basement membrane.) to generate an excess of androgen via LH (LH). An overabundance of androgen synthesis impedes the regular development and maturation of follicles. As a consequence, follicular development is halted andPCOSMmanifests; hypoovulation or anovulation ensues, ultimately culminating in the development of polycystic ovary syndrome.^[48]^

### 5.2. Complications: PCOS can cause metabolic dysfunction

Hyperandrogenism is regarded as a significant characteristic of PCOS. Due to the significant role that hyperandrogenism plays in the development of metabolic disorders associated with PCOS, the metabolic syndrome induced by PCOS is dominated by androgens. A variety of metabolic syndromes, including those affecting the liver, lipids, and central nervous system, can result.

#### 5.2.1. Effects of PCOS on fat metabolism

Clinical research has demonstrated that women diagnosed with PCOS have a higher prevalence of obesity in general. Additionally, PCOS patients have thicker intraperitoneal and mesenteric fat pools than healthy women, and circulating androgen levels are positively correlated with the thickness of the intraperitoneal fat pool in these individuals. Furthermore, PCOS can result in excessive fat accumulation, which suggests that PCOS does influence fat metabolism.^[49]^ Additionally, anti-androgen compound flutamide reduced abdominal fat reserves in women with PCOS during long-term treatment.^[50]^ However, the molecular mechanism underlying chronic androgen exposure-induced abdominal obesity remains mainly unknown. Research conducted in preclinical settings using female rodents has indicated that an excess of androgens could potentially hinder leptin’s capacity to stimulate energy expenditure, thereby potentially facilitating the accumulation of visceral fat.^[51]^ However, additional research is required to clarify the fundamental mechanisms that underlie the impact of hyperandrogenism on patterns of fat accumulation.

#### 5.2.2. Effects of PCOS on liver metabolism

Nonalcoholic fatty liver disease (NAFLD) has been found to be significantly correlated with PCOS, according to numerous studies.^[52]^ Nonalcoholic steatohepatitis (NASH) and cirrhosis are all subtypes of NAFLD, which is a prevalent liver condition encompassing steatosis and cirrhosis, among others. Hepatocellular carcinoma may indeed develop in certain instances of NAFLD. Metabolism-related pathologies, including insulin resistance, obesity, and dyslipidemia, are frequently observed in women with PCOS and contribute to the pathogenesis of NALFD. Hypoandrogenism may increase the risk of NAFLD in these patients, according to recent studies. A case-control study revealed that women with PCOS and hyperandrogenism had a higher risk of developing NAFLD in comparison to healthy women and women without hyperandrogenism, after adjusted for body mass index and IR.^[53]^ Unaffected by age, body mass index, or insulin resistance, an increased free androgen index was also linked to a higher incidence of NAFLD among women with PCOS.^[54]^ In light of this, hyperandrogenism may be regarded as an independent risk factor for the development of NAFLD in women with PCOS, according to the vast majority of the evidence at hand.

Regarding the mechanism by which excessive androgen and the development of NAFLD in women with polycystic ovary syndrome are associated, there remains a dearth of mechanistic research. Elevated androgen levels induced by chronic exogenous human chorionic gonadotropin (hCG) use elicit an imbalance between de novo lipogenesis and mitochondrial b-oxidation in female rats, according to a study. Additionally, this disruption impacts the Peroxisome Proliferator-Activated Receptor (PPAR)-a/b-Srebp1/2-Acc1 axis, resulting in the buildup of adipose tissue in the liver.^[55]^ Research has indicated that hyperandrogenism inhibits the activity of low-density lipoprotein receptor (LDL-R) in the adipose tissue of individuals with PCOS, potentially contributing to the deposition of fat in the liver. It has also been demonstrated that hyperandrogenism inhibits low-density LDL-R expression in the adipose tissue of patients with polycystic ovary syndrome; this effect may promote hepatic fat accumulation.^[56]^ A recent preclinical investigation involving female rodents suggests that prenatal exposure to androgens may modify the PPAR system, which is responsible for liver lipid metabolism changes and an increased risk of liver steatosis and NAFLD.^[57]^ While these results establish a connection between hyperandrogenism and NAFLD, additional preclinical research is required to establish a link between NAFLD and PCOS.

#### 5.2.3. Effects of PCOS on pancreatic metabolism

As previously stated, hyperinsulinemia and insulin resistance are common in women with PCOS, thereby elevating their susceptibility to type 2 diabetes. While considerable research has been conducted on the impact of hyperandrogenism on systemic insulin sensitivity, the specific effects of hyperandrogenism on beta-cell function and insulin release have received relatively little attention. However, some studies suggest that in women with PCOS, an excess of androgens may impair glucose tolerance and beta-cell function. Nonetheless, the precise mechanism through which androgens influence the pancreas has yet to be investigated.^[58]^ The identification of ARs on b-cells through experimental investigations provides support for the notion that androgens might exert a direct influence on the functionality of the pancreas.^[59]^ Compared to control islets, testosterone-treated islets in an in vitro study of isolated rat islets demonstrated impaired glucose-stimulated insulin secretion and mitochondrial dysfunction. Nevertheless, the aforementioned effect was not detected in islets that had been pretreated with the antiandrogen flutamide prior to testosterone treatment.^[60]^ An excess of androgen, which is capable of inducing systemic oxidative stress, may also lead to b-cell dysfunction, according to preliminary studies conducted on female rodents.^[61]^ Nevertheless, the nature of the detrimental impact that testosterone has on b-cell function remains unclear, whether it be direct or indirect. Further investigations established that androgens exert a direct influence on pancreatic function in both males and females.^[62]^ In addition, cultured islets of female rodents and women provided further confirmation of the direct impact of androgens on b-cells. The findings of this research indicated that an overabundance of AR activation in b-cells could potentially result in b-cell failure due to mitochondrial hyperfunction, oxidative damage, and insulin hypersecretion.^[59]^ Through both direct and indirect mechanisms, these results suggest that androgen excess may contribute to b-cell dysfunction in women with PCOS. Further experimental analysis is required, nevertheless, in order to investigate the underlying mechanisms at play

#### 5.2.4. Effects of PCOS on central nervous system metabolism

Moreover, androgens promote metabolic dysfunction in patients with PCOS by interacting with particular brain centers. Melanocortin signaling in the dorsothalamic-macrophage axis (DMH) and communication between hypothalamic nuclei and brown adipose tissue are disrupted by hyperandrogenism, which has a negative impact on central leptin sensitivity and brown adipose tissue thermogenesis mediated by leptin.^[51]^ Metabolic disorders linked to polycystic ovary syndrome may be exacerbated and leptin’s weight-loss properties compromised. Studies have also indicated that excessive prenatal androgen exposure may lead to a higher percentage of Agouti-related protein (AgRP) neurons in the adult brain, potentially influencing energy expenditure and exacerbating obesity-related complications. Co-localization of AgRP and AR in the hypothalamus suggests that androgens via these neurons may modify metabolic homeostasis. Co-localization of AgRP and insulin receptors is additionally diminished by prenatal androgen exposure.^[63]^

In particular, the development of the majority of metabolic and reproductive abnormalities linked to hyperandrogenism appears to be dependent on Androgen receptor (AR) signaling in neurons.^[59]^ Recent studies have shown that the neuroendocrine effects of androgens are significant in the formation of PCOS-related metabolic and reproductive traits. A research investigation that compared and contrasted 3 mouse strains with site-specific AR signal loss, including: induced a PCOS-like phenotype in maturity via chronic postweaning exposure to DHT 1. A mouse line with a global absence of AR signaling, in this mouse model with a global knockout of the AR, widespread metabolic and reproductive dysfunction was observed; however, it did not exhibit PCOS-characteristic obesity or ovarian dysfunction.; 2. A mouse line devoid of AR specifically in neurons, this mouse model with neuron-specific knockout of the AR gene did not significantly affect metabolic or ovarian function, suggesting that AR signaling in the central nervous system plays a limited role in the pathogenesis of polycystic ovary syndrome (PCOS).; 3. A mouse strain with AR inactivation restricted to granulosa cells, This mouse model exhibited obesity, dyslipidemia, polycystic ovarian morphology, and ovulatory dysfunction. the findings suggest that AR signaling deficiency in granulosa cells leads to local androgen metabolism imbalance within ovarian follicles, triggering abnormal estrogen synthesis and increased follicular atresia, ultimately manifesting as obesity, dyslipidemia, and polycystic ovarian morphology.^[64]^ Another study demonstrated that androgen excess induces the development of type 2 diabetes mellitus (T2D) in female mice via specific activation of AR in neurons, which results in hepatic and b-cell insulin resistance, elevated oxidative stress, insulin hypersecretion, and b-cell depletion.^[59]^ Chronic activation of AR in the brain was found to influence peripheral insulin resistance in these mice. These studies collectively indicate that the mechanisms underlying metabolic and reproductive disorders linked to polycystic ovary syndrome are significant and involve the direct impact of androgens on the brain.^[65]^

## 6. Conclusions

This research offers significant contributions by employing bibliometric and visual analysis techniques. An increasing number of articles are published annually on the effects of sex hormones on PCOS, which indicates that sex hormones are gaining greater international attention in the field of PCOS disease and that more academics and clinicians are required to participate. Based on country analysis, the United States and China, being the most active nations in this domain, have forged strong collaborative alliances with both developed and developing countries. However, the analysis of the top ten agencies indicates that intra-country cooperation, such as in China, continues to dominate inter-agency cooperation. This suggests that there is still a lack of international cooperation and the establishment of a worldwide network of collaborative partnerships is premature. Neither of these factors are favorable for the advancement of research concerning the effects of sex hormones on polycystic ovary syndrome. Therefore, we strongly advise that research institutions from various nations conduct more extensive and detailed collaborative investigations in order to facilitate the formation of a global collaborative network for the study of the effects of sex hormones on PCOS. The study by Richard S. Legro and Ricardo Azziz, academic specialists in the study of the effects of sex hormones on PCOS, provides valuable references for clinicians, academics, and medical students and reflects the current emphasis on these topics. Gynecological Endocrinology is a highly regarded journal in which specialists report on the most recent developments and emerging areas of research. Medical student-oriented courses should focus on the most valuable and influential research in the field, as evidenced by the references with the highest number of co-citations.

### 6.1. Shortcomings in past and current research

Previous and current studies have identified hyperandrogenism, insulin resistance, and ovarian dysfunction as core features of PCOS, focusing on the influence of sex hormones on follicular development, GnRH/LH/FSH axis regulation, and metabolic disturbances. These studies have also established associations between PCOS and metabolic syndrome (e.g., obesity, type 2 diabetes), cardiovascular risks, and nonalcoholic fatty liver disease (NAFLD). From the 1990 NIH criteria to the 2013 AES guidelines, ultrasound-based features and hyperandrogenic manifestations have been progressively incorporated into PCOS diagnosis. However, most research has focused on isolated mechanisms (e.g., insulin signaling pathways) or complications (e.g., NAFLD), lacking systemic integration and interdisciplinary perspectives. Although China and the United States are high-output countries, institutional collaborations remain largely domestic (e.g., partnerships between Chinese universities), with limited multinational studies, restricting data sharing and the generalizability of conclusions. Additionally, there is a disconnect between basic research (e.g., AR signaling in pancreatic β-cells) and clinical practice (e.g., personalized treatment protocols), resulting in a lack of targeted intervention strategies for complications. Furthermore, while the Rotterdam criteria are widely adopted, they carry a risk of overdiagnosis (e.g., in PCO patients without hyperandrogenism) and lack dynamic stratification standards based on sex hormone profiles.

### 6.2. Research direction and prospect

Based on past and current research findings, our team proposes that future research directions should focus on the following areas:

1. Deepening molecular mechanism research: Investigate the epigenetic regulatory networks of AR in extraovarian tissues (e.g., liver, pancreas, hypothalamus). Dissect the role of sex hormone interactions with the immune microenvironment (e.g., macrophage polarization, inflammatory cytokines) in PCOS-related chronic low-grade inflammation.
2. Developing precision diagnosis and treatment strategies: Establish a PCOS classification system based on sex hormone subtypes (e.g., free testosterone, sex hormone binding globulin) and metabolic phenotypes to guide personalized therapies. Evaluate the long-term efficacy and safety of novel anti-androgen drugs (e.g., selective AR modulators) for managing metabolic complications.
3. Strengthening international collaboration and data sharing: Build multinational, multicenter cohorts incorporating diverse populations (varying races, ages, and body mass index) to validate the universality of existing conclusions. Integrate multi-omics data (genomics, metabolomics, hormonal profiles) using artificial intelligence to identify early biomarkers of PCOS.
4. Prioritizing long-term health management: Track postmenopausal cardiovascular disease and cancer risks in PCOS patients to explore intervention windows for hormone replacement therapy. Conduct longitudinal studies on the effects of lifestyle interventions (e.g., ketogenic diets, intermittent fasting) on sex hormone balance and ovarian function.

In brief, this research endeavor furnishes critical insights into the domain of sex hormone impacts on PCOS by means of bibliometric and visual evaluation. By doing so, it aids academics, clinicians, and medical students in comprehending influential sources, historical and contemporary research priorities, and prospective developments in PCOS research.

### 6.3. Highlights and shortcomings

This study is the first bibliometric analysis focusing on the field of “the effects of sex hormones on polycystic ovary syndrome (PCOS),” filling a gap in bibliometric research within this field. Utilizing tools such as CiteSpace, VOSViewer, and R-bibliometrix, this study reveals research hotspots (e.g., insulin resistance, hyperandrogenism), development trends, and international collaboration networks (with China and the United States as core collaborating countries) over the past 22 years. It identifies the interdisciplinary characteristics of PCOS research (involving endocrinology, metabolism, immunology, etc) and highlights the current lack of international collaboration, providing data-driven support for future global cooperation. Additionally, this study clarifies the pivotal roles of leading scholars such as Ricardo Azziz and Richard S. Legro, as well as high-impact journals like Fertility and Sterility and Journal of Clinical Endocrinology & Metabolism, offering foundational references for subsequent research.

Nevertheless, this research also possesses certain drawbacks or deficiencies. Initially, the data was exclusively acquired from the WoSCC database, disregarding other databases in the process, potentially resulting in the exclusion of pertinent studies. Second, although the analysis encompasses a great deal of the research into the effects of sex hormones on PCOS, it is possible that certain particulars were omitted. Furthermore, the preoccupation with publications in the English language might have fostered a prejudice against those written in other tongues. The study period is restricted to articles published until November 24, 2023, thereby excluding any subsequent studies. Bibliometric methods, although offering valuable insights, are currently incapable of analyzing the entirety of a publication’s text and thus may omit certain information. In order to offset these limitations, the research study emphasizes significant research patterns and trends by drawing upon references and publications that experience substantial surges in citations, as well as by conducting keyword analysis to condense popular topics and trends.

### 6.4. Innovation and significance of this study

1. Filling methodological gaps:This study is the first to employ bibliometrics to comprehensively map the interplay between sex hormones and PCOS research, providing scholars with a “knowledge map” that identifies research trends, knowledge clusters, and collaboration opportunities. This framework enables rapid navigation of the field’s intellectual landscape.
2. Promoting interdisciplinary integration:Through dual-map overlay analysis (CiteSpace), we clarified the cross-disciplinary nature of PCOS research, particularly the immune-metabolic axis (e.g., macrophage polarization and insulin signaling crosstalk). We advocate for future studies to integrate emerging fields such as epigenetics and gut microbiome dynamics to unravel PCOS heterogeneity
3. Guiding clinical practice:Our analysis identifies hyperandrogenism and its metabolic complications (e.g., NAFLD, β-cell dysfunction) as enduring research hotspots. These findings underscore the urgency to: 1. Develop targeted therapies (e.g., AR antagonists, selective AR modulators); 2. Optimize personalized diagnostic frameworks based on sex hormone profiles (e.g., free testosterone/SHBG ratios); 3. Prioritize long-term health monitoring for PCOS-related cardiovascular and oncological risks.1.

## Acknowledgments

Thanks to all the authors who contributed to the study of the effects of sex hormone profiles in women with polycystic ovary syndrome

## Author contributions

**Conceptualization:** bo li, Shuang Li, Zibo Duan, Hui Yu, Yan Zhou, Xiaohua Lin.

**Data curation:** bo li, Shuang Li, Hui Yu.

**Formal analysis:** bo li, Zibo Duan, Hui Yu.

**Investigation:** Yan Zhou.

**Methodology:** bo li.

**Visualization:** bo li, Shuang Li, Zibo Duan.

**Writing – original draft:** bo li, Shuang Li, Hui Yu.

**Writing – review & editing:** bo li, Hui Yu, Yan Zhou, Xiaohua Lin.

## Supplementary Material

**Figure:**